# Cytotoxic Effect of Trabectedin In Human Adrenocortical Carcinoma Cell Lines and Primary Cells

**DOI:** 10.3390/cancers12040928

**Published:** 2020-04-09

**Authors:** Andrea Abate, Elisa Rossini, Sara Anna Bonini, Martina Fragni, Deborah Cosentini, Guido Albero Massimo Tiberio, Diego Benetti, Constanze Hantel, Marta Laganà, Salvatore Grisanti, Massimo Terzolo, Maurizio Memo, Alfredo Berruti, Sandra Sigala

**Affiliations:** 1 Section of Pharmacology, Department of Molecular and Translational Medicine, University of Brescia, 25123 Brescia, Italy; a.abate005@unibs.it (A.A.); e.rossini013@unibs.it (E.R.); sara.bonini@unibs.it (S.A.B.); martina.fragni@gmail.com (M.F.); maurizio.memo@unibs.it (M.M.); 2 Oncology Unit, Department of Medical and Surgical Specialties, Radiological Sciences, and Public Health, University of Brescia at ASST Spedali Civili di Brescia, 25123 Brescia, Italy; deborah.cosentini@gmail.com (D.C.); martagana@gmail.com (M.L.); grisanti.salvatore@gmail.com (S.G.); alfredo.berruti@gmail.com (A.B.); 3 Surgical Clinic, Department of Clinical and Experimental Sciences, University of Brescia at ASST Spedali Civili di Brescia, 25123 Brescia, Italy; guido.tiberio@unibs.it; 4 Thoracic Surgery Unit, ASST Spedali Civili of Brescia, 25123 Brescia, Italy; drbenettidiego@gmail.com; 5 Klinik für Endokrinologie, Diabetologie und Klinische Ernährung, Universitätsspital Zürich, 8091 Zurich, Switzerland; Constanze.Hantel@usz.ch; 6 Medizinische Klinik und Poliklinik III, University Hospital Carl Gustav Carus Dresden, 01307 City, Germany; 7 Department of Clinical and Biological Sciences, University of Turin, Internal Medicine 1, San Luigi Gonzaga Hospital, 10043 Orbassano, Italy; massimo.terzolo@unito.it

**Keywords:** trabectedin, adrenocortical carcinoma, cytotoxicity, in vitro, cell lines, primary cell cultures

## Abstract

Mitotane is the only drug approved for the treatment of adrenocortical carcinoma (ACC). The regimen to be added to mitotane is a chemotherapy including etoposide, doxorubicin, and cisplatin. This pharmacological approach, however, has a limited efficacy and significant toxicity. Evidence indicates that ACC seems to be sensitive to alkylating agents. Trabectedin is an anti-tumor drug that acts as an alkylating agent with a complex mechanism of action. Here, we investigated whether trabectedin could exert a cytotoxic activity in in vitro cell models of ACC. Cell viability was evaluated by MTT assay on ACC cell lines and primary cell cultures. The gene expression was evaluated by q-RT-PCR, while protein expression and localization were studied by Western blot and immunocytochemistry. Combination experiments were performed to evaluate their interaction on ACC cell line viability. Trabectedin demonstrated high cytotoxicity at sub-nanomolar concentrations in ACC cell lines and patient-derived primary cell cultures. The drug was able to reduce /β catenin nuclear localization, although it is unclear whether this effect is involved in the observed cytotoxicity. Trabectedin/mitotane combination exerted a synergic cytotoxic effect in NCI-H295R cells. Trabectedin has antineoplastic activity in ACC cells. The synergistic cytotoxic activity of trabectedin with mitotane provides the rationale for testing this combination in a clinical study.

## 1. Introduction

Adrenocortical carcinoma (ACC) is a rare and aggressive tumor characterized by an estimated incidence of 0.7–2.0 cases/million people per year and an overall 5 year survival rate less than 15% in patients with metastatic disease [1]. To date, radical surgery at experienced centers still remains the only potential curative treatment for patients with early disease stage and those with locally advanced ACC responding to neoadjuvant treatment. However, 30–70% of radically operated patients recur within two years, often with metastatic disease [1]. Clinical manifestations of ACC can be consequences of adrenal hormone excess (functional tumors) or growing abdominal masses. Standard systemic therapies for patients with advanced ACC are mitotane and chemotherapy [2]. The dichlorodiphenyl trichloroethane derivative mitotane represents the only drug that has been approved for the treatment of ACC for many decades, although its mechanism of antineoplastic activity is not fully understood [3] Clinical evidence shows that the efficacy of mitotane is strictly dependent on the attainment and the maintenance over time of circulating blood levels within the 14–20 mg/dL range [2]. Due to the pharmacokinetic characteristics of mitotane, this value is usually reached in about 2–3 months [4], and this long latency could impair, at least, in part, the efficacy of the treatment. The standard chemotherapy regimen to be added to mitotane as first-line treatment for advanced ACC is the combination of etoposide, doxorubicin, and cisplatin (EDP) [5,6]. The EDP-Mitotane (EDP-M) regimen, however, has a limited efficacy, and its administration is burdened by significant toxicity. Moreover, no efficacious second line therapies are available as of yet [7,8]. Therefore, new treatment strategies are needed. DNA alkylation appears to be a critical point for inducing cytotoxicity in the ACC; indeed, cisplatin is a fundamental component of the EDP-M scheme. Furthermore, results obtained both in vitro [9] and in vivo [10] support the antineoplastic activity of another alkylating agent such as temozolomide against ACC. Trabectedin (ET-743) is a powerful anti-tumor drug isolated from the Caribbean tunicate *Ecteinascidia turbinata*. The compound is approved for the treatment of soft tissue sarcoma (STS) and relapsed platinum-sensitive ovarian cancer (OC). Although trabectedin acts as an alkylating agent, its mechanism of action is more complex than expected. Indeed, the drug interferes with the minor groove, binding the guanine to the exocyclic N2 amino group [11], modifying the recognition of GC-enriched sequences of transcriptional factors often involved in the oncogene transcription. It has been observed that trabectedin interferes with FUS-CHOP and EWS-CHOP fusion proteins, inhibiting their ability to induce the transcription of different oncogenes [12,13] Furthermore, in vitro studies demonstrate that trabectedin downregulates the Wnt/β-catenin pathway in cell cultures of biliary tract adenocarcinoma [14]. Finally, trabectedin was shown to modulate the activity of immune cells in the tumor microenvironment, reducing the production of pro-inflammatory mediators (for a review, see [11]). These assumptions led us to hypothesize that trabectedin could exert a cytotoxic effect in ACC as well. To explore this issue, we took advantage of the experimental in vitro model of ACC cell lines as well as patient-derived ACC primary cell cultures.

## 2. Results

### 2.1. Trabectedin Induced Cytotoxicity in ACC Cell Lines

**Figure 1:**
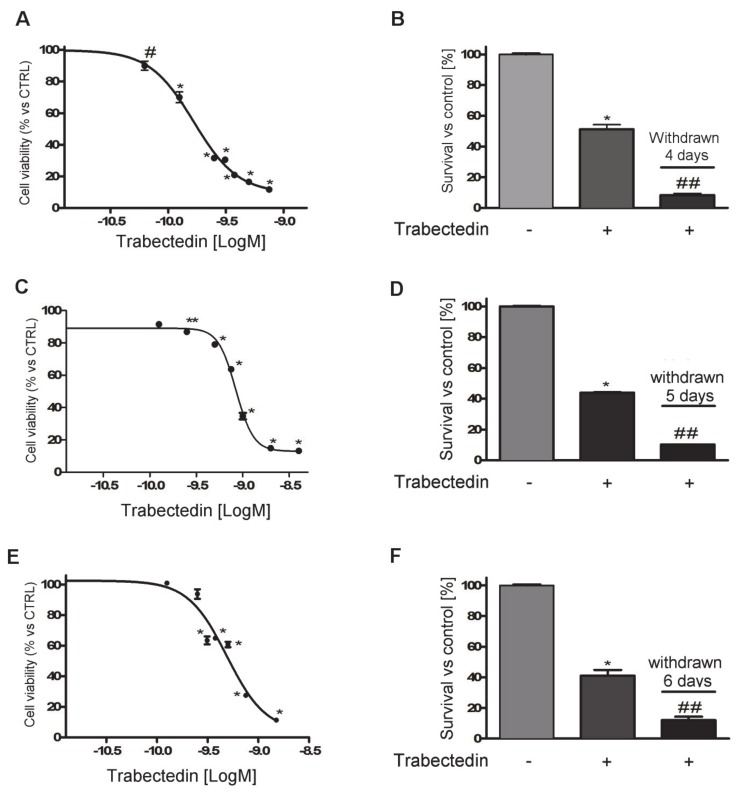
Cytotoxic effect of trabectedin in adrenocortical carcinoma (ACC) cell models. (**A**) Concentration–response curve of trabectedin-induced inhibition of cell viability of in NCI-H295R cells. Cells were treated with increasing concentrations of trabectedin (0.0625–0.75 nM) for 4 days. (**B**) Cytotoxic effect lasted after trabectedin withdrawn. Cells were treated with the trabectedin IC50 (0.15 nM) for 4 days, then trabectedin was withdrawn from medium, and cells were kept in culture for a further 4 days. (**C**) MUC-1 cells were treated for 5 days with increasing concentrations (0.125–2.0 nM) of trabectedin. (**D**) MUC-1 cells were treated with 0.80 nM trabectedin for 5 days, then trabectedin was withdrawn from medium, and cells were kept in culture for a further 5 days. (**E**) The sub-clone HAC-15 cells were treated for 6 days with increasing concentrations (0.125–1.5 nM) of trabectedin. (**F**) HAC-15 cells were treated with 0.50 nM trabectedin for 6 days, then trabectedin was withdrawn from medium, and cells were kept in culture for a further 6 days. Cell viability was analyzed by MTT assay. Results are expressed as percent of viable cells vs. untreated cell ± SD; * *p* < 0.0001 vs. control; # *p* < 0.001 vs. control; ** *p* < 0.01 vs. control; ## *p* < 0.0001 vs. trabectedin-treated cells.

Exposure of NCI-H295R cells to increasing concentrations of trabectedin (0.0625–0.750 nM) for four days led to a concentration-dependent reduction of cell viability. Sigmoidal concentration-response function was applied to calculate the IC50 value of trabectedin, which was 0.15 nM (95% confidence interval (CI): 0.13–0.17nM). The drug was highly active in inducing cytotoxicity, as its efficacy reached about 90% at the highest concentration tested (Figure 1A).

The cytotoxic effect of trabectedin induced DNA fragmentation (Figure S1) and apoptotic cell death (Figure S2). Cells were then plated and cultured in complete medium added with 0.15 nM trabectedin. Cell viability was assessed at four days of treatment, then the drug was withdrawn, and cells were kept in a drug-naive complete medium to evaluate whether the trabectedin cytotoxic insult was a long-lasting effect. Results show that trabectedin treatment induced cell damage that also progressed in the absence of the drug (Figure 1B).

The cytotoxic effect of trabectedin was then studied in other ACC experimental cell line models. As shown in Figure 1, trabectedin exerted a cytotoxic effect in other ACC cell line models as well, although with different sensitivity and accordingly with their different phenotype. Indeed, as indicated in the Methods section, HAC-15 is a subclone of NCI-H295R, while MUC-1 is an EDP-M resistant cell line recently established. Concentration–response curves of trabectedin in MUC-1 and HAC-15 are reported in Figure 1C,E. Analysis of the curves allowed the evaluation of the respective IC50, which was 0.80 nM (95% CI: 0.77–0.83 nM) in MUC-1 cells and 0.50 nM (95% CI: 0.30–0. 82 nM) in HAC-15 cells. In line with results obtained in NCI-H295R cells, trabectedin induced cell damage, leading to cell death that continued in drug-withdrawn conditions (Figure 1D,F). Figure S3 reports results obtained with SW13 cells, which is of adrenal origin, but it has been suggested to be a small cell carcinoma. These cells are also sensitive to the cytotoxic effect of trabectedin, and the IC50 was 0.098 nM (95% CI: 0.0093–0.104 nM). When cells were exposed to the IC50 trabectedin for three days and then transferred in drug-free medium, the cytotoxic insult elicited by trabectedin induced cell death.

### 2.2. Trabectedin-Induced Cytotoxicity in ACC Primary Cell Cultures

**Figure 2:**
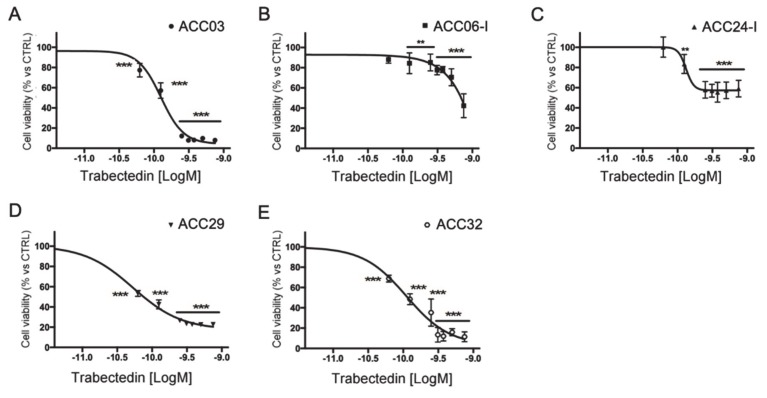
Cytotoxic effect of trabectedin in primary cell cultures derived from ACC patients. Cells were treated with increasing concentrations of trabectedin (0.0625 nM–0.75 nM) for four days. Cell viability was analyzed by MTT assay. Results are expressed as percent of viable cells vs. untreated cells ± SD; ** *p* < 0.001; *** *p* < 0.0001. (**A**): ACC03 primary cell culture; (**B**): ACC06-I primary cell culture; (**C**): ACC24-I primary cell culture; (**D**): ACC29 primary cell culture; (**E**): ACC32 primary cell culture.

Primary cell cultures were prepared from tissue samples obtained from ACC patients who underwent surgery, as described in the Methods section. Trabectedin exerted a concentration-dependent reduction of human ACC primary cell viability (Figure 2); however, as expected, due to the different patient tumor stage and tumor cell characteristics, ACC primary cells displayed a different drug sensitivity. 

Table 1 reports the in vitro efficacy of trabectedin in ACC primary cultures, measured as percentage of maximum cytotoxic effect, and the trabectedin IC50 for each cell culture. In particular, ACC03, ACC29, and ACC32 displayed the higher sensitivity, as the trabectedin-induced cytotoxicity was over 80% compared to untreated cells, with the IC50 that was within low nanomolar concentrations (range: 0.08–0.13 nM).

### 2.3. Trabectedin Enhanced Cytotoxicity Induced by Mitotane in NCI-H295R Cell Line

**Figure 3:**
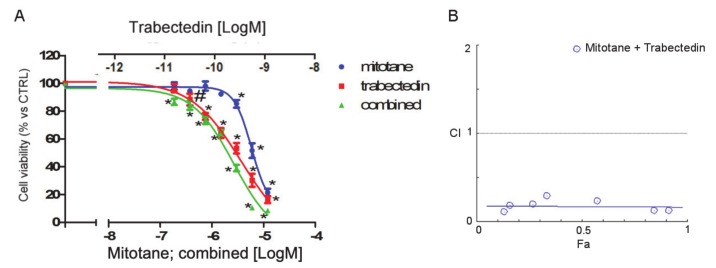
Effect of the trabectedin/mitotane combination on NCI-H295R cell viability. Cells were treated with increasing concentrations of trabectedin and mitotane alone or in combination at fixed concentration molar ratios (trabectedin:mitotane; 1:10000 molar ratio for 4 days. Cell viability was measured by MTT. (**A**) Concentration–response curves. Cells were exposed to increasing concentrations of trabectedin and mitotane alone or in combination at fixed concentration trabectedin:mitotane = 1:10,000 molar ratio. Results are expressed as percent of viable cells vs. untreated cell ± SD. (**B**) Combination index plot. Dose and effect data obtained were converted to Fa values and analyzed with CompuSyn software. # *p* < 0.01; * *p* < 0.0001.

As mitotane is the reference drug for the treatment of ACC, to evaluate whether trabectedin could enhance the cytotoxicity of mitotane in NCI-H295R cells, a combined treatment was performed, applying the Chou-Talalay approach (Figure 3). 

The concentration–response of each drug and of the combined treatment is reported in Figure 3A. The trabectedin/mitotane combined treatment in NCI-H295R cells induced a synergistic cytotoxic effect compared to each single compound. Results obtained were converted to Fa values and analyzed with CompuSyn software and the combination index plot, and the synergistic effect is shown in Figure 3B. The combination index value was <1 at each drug combination tested, suggesting a mainly synergic effect.

### 2.4. Trabectedin Affects β-catenin Localization in NCI-H295R Cell Line

**Figure 4:**
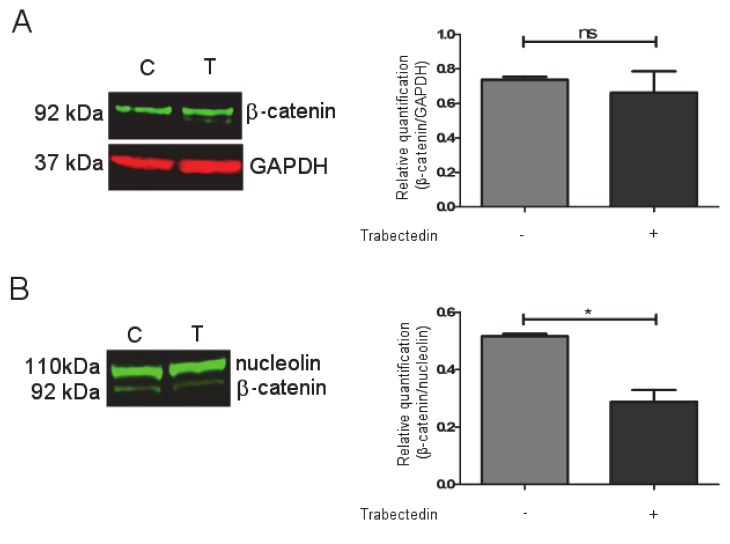
Effect of trabectedin on the β -catenin expression by Western blot (WB) technique. NCI-H295R cells were exposed to 0.15 nM of trabectedin for 3 days. (**A**) A representative WB on total lysate is shown. (**B**) A representative WB on nuclear protein fraction is shown. * *p* < 0.01. ns: not significant.

It was shown in the cholangiocarcinoma model that the Wnt/β catenin pathway could be a target of trabectedin [14]. Based on these findings, we investigated whether trabectedin could influence β-catenin expression and function in NCI-H29R cells. Western blots (Figure S4) were performed on trabectedin-exposed NCI-H295R cells, and results demonstrated that the total protein expression of β-catenin was not modified by drug exposure (Figure 4A), while, when the nuclear/cytoplasm subcellular localization was investigated, we observed that, after three days of trabectedin treatment at its IC50 value, the β-catenin nuclear concentration was reduced by 44 ± 7.3% in treated cells compared to vehicle-treated cells. (Figure 4B).

**Figure 5:**
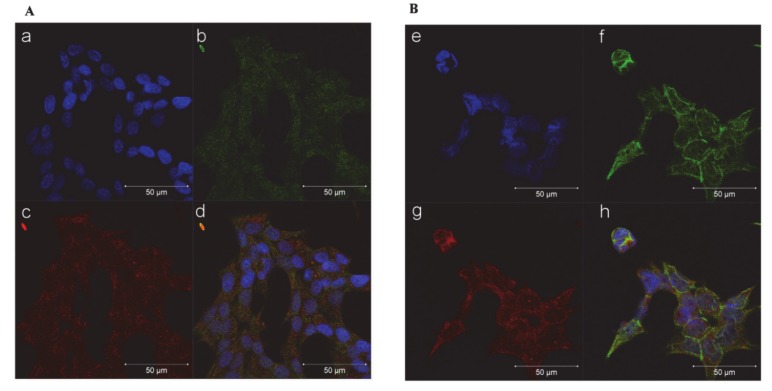
Trabectedin exposure affects the subcellular localization of β-catenin in NCIH295R cells. Cells were treated with 0.15 nM trabectedin for 3 days. Untreated (**A**) and trabectedin (**B**) treated cells were analyzed for β-catenin localization following by incubation with Hoechst for nuclear staining. Panels a, e: Hoechst; panels b, f: β-catenin; panels c, g: constitutive proteasome subunit PSMB5; panels d, h: merge. The scale bar of 50 µm is automatically inserted by the software ZEN Black.

A representative figure of subfraction proteins is reported in Figure S5. Furthermore, immunocytochemistry experiments performed in the NCI-H295R cells and reported in Figure 5 indicate that, after 72 h of trabectedin treatment, the co-localization of β-catenin with the constitutive proteasome subunit PSMB5 was increased, suggesting that the drug induced a translocation of β-catenin from the nucleus to the cytoplasm, in particular to the proteasome, leading to its degradation. Indeed, β-catenin translocation from nucleus to cytoplasm is necessary to turn off its transcription-factor activity and to direct the protein to proteasomal degradation [15]. A time course is shown in Figure S6.

### 2.5. Trabectedin Affects the Expression of β-catenin Target Genes

**Figure 6:**
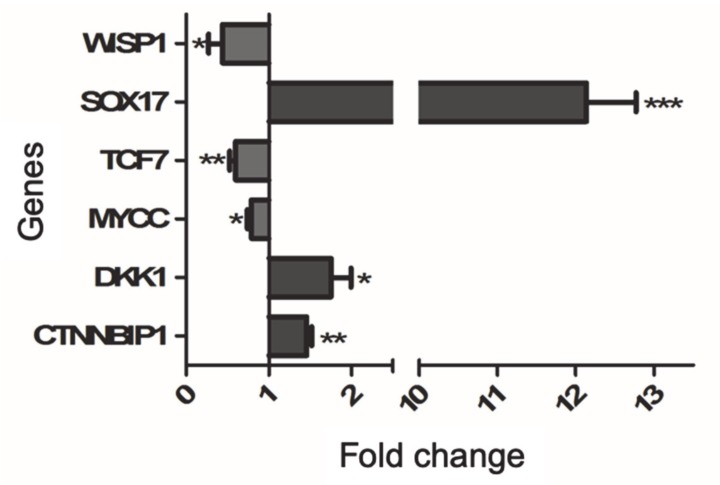
Validation of selected Wnt/β-catenin pathway by qRT-PCR in trabectedin-treated NCI-H295R cells. RNA was extracted and retro-transcribed as described in Materials and Methods. Genes were measured by q-RT-PCR using SYBR Green as fluorochrome. Results are presented as fold change ± SEM; * *p* < 0.01; ** *p* < 0.05; *** *p* < 0.001.

Whether the reduction of β-catenin nuclear localization can affect the gene expression regulator was investigated, taking advantage of the specific Wnt/β-catenin pathway signaling RT2 profiler array, as described in Methods. Representative genes modified by trabectedin treatment were then validated by qRT-PCR analysis in vehicle-treated and trabectedin-treated NCI-H295R cells. In particular, we selected target genes known to be critical in cancer development, namely TCF7, DKK1, WISP1, MYCC, and SOX17. Trabectedin exposure significantly increased the expression of genes that encode proteins involved in the reduction of Wnt activity, i.e., CTNNBIP1, DKK1, and SOX17, while the protoncogene MYCC and genes activating the Wnt pathway, namely TCF7 and WISP1, were significantly reduced in NCI-H295R cells after exposure to trabectedin (Figure 6).

## 3. Discussion

Trabectedin displays a manifold mechanism of action that impacts tumor cell biology and tumor microenvironments. The alkylating activity together with the inhibition of transcription, due to the interaction with RNA polymerase II and DNA repair proteins, appear to be the hallmarks of the antiproliferative activity of this drug [16]. 

The present study was undertaken to investigate the trabectedin antitumor activity in in vitro experimental cell models of ACC. The results revealed that trabectedin is highly cytotoxic at sub-nanomolar concentrations both in ACC cell lines and primary cell cultures derived from patients who underwent surgery for ACC. Trabectedin showed high potency and efficacy, reaching a reduction of cell viability of about 90% compared to untreated cells. These results are in line with those obtained in soft tumor sarcoma (STS) cells [17] and in ovary carcinoma cell lines [18]. Additionally, the IC50 obtained in ACC cells was in the same range of the above mentioned cell lines. It was noteworthy that the cytotoxic damage induced by trabectedin showed an increasing trend when ACC cell lines were withdrawn from the drug and maintained in the normal growth medium. These data suggest that the damage induced by trabectedin was not repaired upon withdrawal, and this is consistent with the trabectedin capability to inhibit the transcription-dependent nucleotide excision repair pathways, leading to p53-independent apoptosis [19]. 

It was also noteworthy that the sensitivity of tumor cells to trabectedin antineoplastic activity varied consistently among the different cell lines evaluated, as it was at maximum against NCI-H295R and less evident against MUC-1, which was derived from an ACC patient whose tumor was resistant to the standard EDP-M scheme. This would imply that trabectedin could be efficacious when administered upfront, while its efficacy could decrease if the drug was employed as a second line approach in EDP-M treated patients. This hypothesis is strengthened by results obtained with the primary cell cultures. Indeed, ACC06-I and ACC24-I cells established from metastases of EDP-M pretreated patients displayed a limited sensitivity to trabectedin with a cytotoxic efficacy around 50%, while ACC03 and ACC32 belonging to untreated local relapse and primary ACC, respectively, were highly susceptible to the trabectedin cytotoxic effect with almost no alive cells left after four days of trabectedin exposure. It should be noted, however, that a degree of variability to trabectedin did exist among patients, as ACC29 cells were derived from a patient that underwent four EDP-M cycles before surgery, but cells still maintained a good response, reaching about 80% of cytotoxicity when they were exposed to the highest trabectedin concentration used. Finally, the SW13 cell line, probably belonging from a small cell carcinoma of adrenal, was highly sensitive to the trabectedin cytotoxic effect.

The observation of the in vitro synergic cytotoxic effect when NCI-H295R cells were treated with the trabectedin and mitotane combination has an interesting potential clinical implication, since the therapeutic potential of the combination can be obtained at low doses of each drug, thus leading to more efficacious therapeutic approaches and ameliorating the toxicity profile. The pharmacometabolic interactions between the two drugs could be a limit of their association in clinics, as mitotane is known to be a CYP3A4/5 inducer, and trabectedin is extensively metabolized by the CYP3A4 (www.micromedexsolutions.com, accessed 10 October 2019). It should be underlined, however, that a limit of the mitotane therapy is its latency of action due to the long half-life that allows the achievement of the initial response after about three months [4]. This period could be critical, especially for patient with advanced disease. It could thus be speculated that a sequential/combined trabectedin plus mitotane treatment could be of clinical interest with the advantage of a prompt cytotoxic effect, which could be improved when the two drugs are overlapping. Periodical evaluation of trabectedin plasma concentration could be indicated to highlight the possible pharmacometabolic induction of trabectedin by the mitotane. 

The Wnt/β-catenin pathway activation in ACC is notoriously a major tumor driver in the pathogenesis of ACC [20] and a mechanism of ACC resistance to modern immunotherapy [21,22]. NCI-H295R cells harbor an activating point mutation in the β-catenin gene CTNNB1 that modifies the Ser45 of exon 3, leading to enhanced Wnt/β-catenin transcriptional activity and increased nuclear localization [23,24]. Despite the constitutive activation of the CTNNB1 gene, however, the Wnt/β-catenin pathway in the NCI-H295R cell model retains, at least partially, the capability to respond to exogenous regulatory stimuli. This was shown to be induced by progesterone in previous studies of our group [25,26] as well as in a study testing the Wnt/β-catenin inhibitor PNU-7654 [24].

It was noteworthy that Peraldo-Neia [14] recently showed, in preclinical cell models of cholangiocarcinoma, that trabectedin is able to inhibit the Wnt/β-catenin pathway, leading to downregulation of MYCC expression, proliferation inhibition, and apoptosis enhancement. Here, we provided preliminary evidence that trabectedin treatment reduced the nuclear localization of β-catenin in NCI-H295R cells and increased the co-localization of β-catenin with cytoplasmatic proteasomal protein markers, thus suggesting a proteosomal degradation. The reduction of β-catenin nuclear localization found a functional effect in the modification of the expression of some target genes involved in the activity and in the regulation of this pathway. We provided indications that trabectedin leads to the increased expression of CTNNBIP1 (β-catenin-interacting-protein-1) and DKK1 (Dickkopf-related protein) that inhibit [27] and antagonize [28,29] the Wnt signaling pathway, respectively. The apparent discrepancy of the DKK1 mRNA increase found its rationale in the evidence that alkylating drugs directly stimulate DKK1 gene expression [30]. Thus, the results we observed may be the balance between the reduction of DKK1 mRNA expression induced by a decrease of nuclear β-catenin [31] and the mRNA increase in response to the alkylating action of trabectedin. Moreover, we observed a strong increase of the gene encoding the transcription factor SOX17, a Wnt signaling antagonist [32,33,34], and reduction of TCF7 gene expression, which is involved in the trabectedin-induced reduction of cell viability [24]. The Wnt downstream regulator WISP1 (WNT1-inducible signaling pathway protein-1) (for a review, see: [35]) and MYCC gene expressions (reviewed in [36]) were also reduced. 

We are aware that gene expression does not linearly correlate with the protein translation, and that these data need confirmation with the protein expression. Nevertheless, our results begin shedding light on the possible involvement of this crucial pathway in the trabectedin mechanism of action, strongly indicating that it is even more complex than hitherto demonstrated.

## 4. Materials and Methods 

### 4.1. Cell Lines

NCI-H295R, HAC-15, and SW13 cell lines were purchased from ATCC (American Type Culture Collection, Manassas, VA, USA.) and maintained as suggested. MUC-1 cells were kindly given by Dr. Hantel and were maintained according to [37]. Media and supplements were supplied by Sigma Aldrich Italia, (Milano, Italy). NCI-H295R cells are the worldwide known in vitro ACC model [38]. HAC-15 cells were derived from the NCI-H295R cell line and retained the ACTH-sensitivity [39]. The SW13 cell line was established from a small cell carcinoma in the adrenal cortex, but its histopathologic characteristics are still under investigation [40]. Finally, MUC-1 cells were derived from a neck metastasis of an EDP-M treated patient [37].

### 4.2. Primary Cultures

The ACC primary cultures belonged to five patients who underwent surgical resection of primary or metastatic ACC. One of them (ACC29) presented a cortisol-secreting tumor, while others were non-secreting tumors. The clinical characteristics of patients are summarized in Table 2. 

The project was approved by the local Ethics Committee (NP 1924) and informed consent was signed by each patient enrolled in the study. Primary ACC cultures were obtained according to the previously published procedure [41]. The adrenal origin of primary cells was confirmed by the positivity to the steroidogenic factor 1 gene expression [37,42].

### 4.3. Cell Viability Assay

Cell viability was evaluated by 3-(4,5-Dimethyl-2-thiazol)-2,5-diphenyl-2H-tetrazolium bromide (MTT) dye reduction assay according to the manufacturer protocol (Sigma Aldrich). Drug- or vehicle-treated cells were incubated with MTT dye (at final concentration of 0.5 mg/mL) and solubilized by DMSO. Absorbance was measured by a spectrophotometer at 570 nm.

### 4.4. Cell Treatments

Cells were plated in 96 wells-plates at the concentration of 6 × 103 cells/well for NCI-H295R and HAC-15 and 5 × 103 cells/well for SW13, MUC-1, and ACC primary cultures. For the concentration–response curves, cells were exposed to increasing concentrations of trabectedin for different times, according to the respective doubling time. NCI-H295R cell lines and ACC primary cultures were exposed to trabectedin (0.0625–0.75 nM) for 4 days, while SW13 cell treatment lasted for 3 days. HAC-15 cells were treated with increasing concentrations of trabectedin (0.125–1.5 nM) for 6 days. Finally, MUC-1 was exposed to trabectedin (0.125–2.0 nM) for 5 days.

### 4.5. Drug Combination Experiments

Trabectedin and mitotane combination experiments were performed to evaluate their interaction on NCI-H295R cell viability, according to the Chou and Talaly method [43]. NCI-H295R was treated for 4 days with trabectedin (0.018–1.2 nM) and mitotane (0.18–12 µM) alone or in combination with a fixed ratio (trabectedin/mitotane = 1/10,000), as recommended for the most efficient data analysis [44]. Cells were analyzed for cell viability using MTT. Data were then converted to fraction affected (Fa, range from 0 to 1 where Fa = 0 indicating 100% of cell viability and Fa = 1 indicating 0% of cell viability) and analyzed using the CompuSyn software (ComboSyn inc. Paramus, NJ, USA) to calculate the combination index (CI), the CI value < 0.9 being an indication of synergism, CI = 09–1.1 an indication of additive effect, and CI > 1.1 an indication of antagonism.

### 4.6. Quantitative RT-PCR (qRT-PCR)

Total RNA was extracted from cells by RNAeasy kit (Qiagen, Milano, MI, Italy), and 1 μg was transcribed into cDNA using murine leukemia virus reverse transcriptase (Promega Italia, Milano, MI, Italy). Gene expression was evaluated by qRT-PCR (ViiA7, Applied Biosystems, Milano, Italy) using SYBRGreen as fluorochrome, as already described [45]. Sequences of oligonucleotide primers used were reported in Table 3. 

Reactions were performed under the following conditions: 1 cycle at 95 °C for 10 min, 40 cycles at 95 °C for 15 s, 62 °C for 1 min. Differences of the threshold cycle (Ct) values between the β-actin housekeeping gene and the gene of interest (ΔCt) were then calculated as an indicator of difference in the amount of mRNA expressed [46].

### 4.7. WNT/β-catenin Pathway Gene Profile

Total RNA was extracted from vehicle-treated NCI-H295R cells and from cells treated with the IC50 of trabectedin for 4 days using RNAeasy kit (Qiagen). cDNA was synthesized from 0.5 μg RNA using RT2 First Strand Kit (Qiagen), and the mixture was added into a 96-wells WNT pathway signaling RT2 profiler array according to the manufacturer’s instructions (Qiagen). Thermal cycling was performed using ViiA7 with an initial denaturation at 95 °C for 10 min, 40 cycles at 95 °C for 15 s, and 60 °C for 1 min. Qiagen’s online web analysis tool was used to calculate the fold change in trabectedin-treated vs. untreated cells by determining the ratio of mRNA levels to control values using the Δ threshold cycle (Ct) method (2−ΔΔCt). All data were normalized to an average of five housekeeping genes. Validation of selected genes was performed by q-RT-PCR as previously described.

### 4.8. Western Blot

NCI-H295R cells were treated with IC50 value of trabectedin for 4 days. Whole cell lysates were prepared in ice-cold buffer with protease and phosphatase inhibitor cocktails (Roche, Milano, Italy) as previously described [25]. Fractionated cell lysates were prepared according to [47]. Equal amounts of proteins were separated by electrophoresis on a 4–12% NuPAGE Bis-Tris Gel System (Life Technologies, Milano, Italy) and electroblotted to a nitrocellulose membrane. Primary and secondary antibodies are listed in Table 4. Signal was detected and quantified with the Odyssey® CLx Infrared Imaging System (LI-COR Biosciences, Lincoln, NE, USA).

### 4.9. Immunofluorescence

Cells were grown onto 12 mm poly-L-lysine coated coverslips and treated with IC50 value of trabectedin for 3 days. Cells were then fixed in ice-cold methanol for 20 min and permeabilized with 0.2% Triton X-100 in PBS for 1 h. Nonspecific binding was blocked by incubation in phosphate-buffered saline (PBS) containing 0.2% Triton X-100 and 1% of bovine serum albumin (BSA) for 5 min. Cells were then incubated overnight at 4 °C with rabbit monoclonal antibody against human β-catenin primary antibody and with mouse monoclonal antibody against human PSMB5. After extensive washes, the Alexa Fluor488 anti-rabbit secondary antibody (Life Technologies) was applied for 1 h at room temperature, followed by counterstaining with Hoechst (Sigma Aldrich) for 5 min. After rinsing in PBS, coverslips were mounted using FluorPreserveTM Reagent, and cell staining was detected using a Zeiss LSM 510 META confocal laser-scanning microscope (Carl Zeiss AG, Oberkochen, Germany). Zen software was used for image analysis and processing.

### 4.10. Chemicals

Trabectedin was kindly given by Pharma Mar S.A. (Madrid, Spain), dissolved in DMSO, and stored at –20 °C in 10 mM aliquots. Mitotane was supplied by Selleckchem Chemicals (DBA Italia, Milano, Italy), dissolved in DMSO, and stored at –80 °C in 200 mM aliquots.

### 4.11. Statistical Analysis

Statistical analysis was carried out using GraphPad Prism software (version 5.02, GraphPad Software, La Jolla, CA, USA). One-way ANOVA with Bonferroni׳s correction was used for multiple comparisons. Unless otherwise specified, data are expressed as mean ± SD or SEM of at least three experiments run in triplicate. *p* values < 0.05 were considered statistically significant.

## 5. Conclusions

Our results indicate that trabectedin, at concentrations superimposable to those employed in in vitro experimental models of STS and ovary carcinoma, exert a cytotoxic effect in different ACC cell models, and the effect persists upon drug withdrawal. These findings together with the high lipophilicity of trabectedin and the lyophilic milieu of these tumors suggest that the drug may accumulate in ACC cells and could be efficacious in treating ACC patients. This point, however, is speculative and needs to be extensively studied in a dedicated prospective clinical trial. 

Finally, our data on the inhibitory effect of trabectedin on Wnt/β-catenin are interesting due to the major role of this pathway in proliferation and resistance to antineoplastic agents of ACC cells [21,22,48].

## Figures and Tables

**Table 1:** Effects of trabectedin in ACC primary cultures.

Primary Culture Identification	IC50 (95% Confidence Interval)	Maximum Effect
ACC03	0.13 nM (0.12 nM to 0.14 nM)	92 ± 0.63%
ACC06-I	ambiguous	57 ± 11.8%
ACC24-I	0.13 nM (0.10 nM to 0.17 nM)	41 ± 8.2%
ACC29	0.053 nM (0.048 nM to 0.58 nM)	77.2 ± 0.5%
ACC32	0.11 nM (0.087 nM to 0.13 nM)	88.6 ± 4.9%

**Table 2:** Clinical and immunohistochemical characteristics of ACC patients.

Primary Culture Identification	Tumor Specimen	Histology	Disease Stage	Hormone Hypersecretion	SF-1 Expression
**ACC03** Male 59 years old	Local relapse of ACC	Mitotic index: 25/50 HPF; Ki67: 20%	Local relapse	No secretion	2+
**ACC06-I** Male 42 years old	Hepatic metastasis	Mitotic index: 18/50 HPFKi67: 20%	Stage IV, multiple metastases	No secretion	2+
**ACC24-I** Female 45 years old	Lung metastasis	Not available	Stage IV, multiple metastases	No secretion	2+
**ACC29** Female 51 years old	Primary ACC	Mitotic index: 2/50 HPF; Ki67: <5%	Stage IV, lung metastases	Cortisol	2+
**ACC32** Male 66 years old	Primary ACC	Mitotic index: >5/50 HPF; Ki67: 20%	Stage II	No secretion	1+

**Table 3:** Sequences of oligonucleotide primers for qRT-PCR.

Gene	Sense	Oligonucleotide Sequence (5′-3′)
Β-ACTIN	F R	TCTTCCAGCCTTCCTTCCTG CAATGCCAGGGTACATGGTG
SF-1	F R	CAGCCTGGATTTGAAGTTCC TTCGATGAGCAGGTTGTTGC
CTNNBIP1	F R	TTGACAACGGTGACAGCACT TCAGGCAAACAGGTGCTCAAC
DKK1	F R	TAGCACCTTGGATGGGTATT ATCCTGAGGCACAGTCTGAT
MYCC	F R	CGTCCTCGGATTCTCTGCTC CTTCGCTTACCAGAGAGTCGCT
SOX17	F R	GGTGTGAATCTCCCCGACAG TAATATACCGCGGAGCTGGC
TCF7	F R	GTAAACAGACCCCCGCCATC GCCCTCCAACCAAGAAACCT
WISP1	F R	ACCGCCCGAGGTACGC AAGGACTGGCCGTTGTTGTA

**Table 4:** Primary and secondary antibodies.

Target	Characteristic	Company	Final Concentration
β-CATENIN	Rabbit mAb	Cell Signaling Technology (Denvers, MA, USA)	6 ng/mL (WB) 15 ng/mL (IF)
GAPDH	Mouse mAb	Santa Cruz Biotechnology (Dallas, TX, USA)	1 µg/mL
C23 (nucleolin)	Rabbit pAb	Santa Cruz Biotechnology (Dallas, TX, USA)	0.2 µg/mL
PSMB5	Mouse mAb	Abcam (Cambridge, UK)	10 µg/mL
Secondary anti-mouse	IRDye 680CW conjugated	LI-COR Biosciences (Linloln, NE, USA)	0.67 µg/mL
Secondary anti-rabbit	IRDye 800CW conjugated	LI-COR Biosciences (Linloln, NE, USA)	0.67 µg/mL
Secondary anti-mouse	Alexa Fuor 555 Conjugated	Immunological Sciences (Rome, Italy)	5 µg/mL
Secondary anti-rabbit	Alexa Fuor 488 Conjugated	Immunological Sciences (Rome, Italy)	5 µg/mL

## Supplementary Materials

The following are available online at ’https://www.mdpi.com/2072-6694/12/4/928/s1, Figure S1 Effect of trabectedin on DNA integrity, Figure S2 Trabectedin promoted apoptotic cell death in NCI-H295R cells, Figure S3 Cytotoxic effect of trabectedin on SW13 cell line, Figure S4: Whole western blots of Figure 4, Figure S5 Representative figure of subfractions proteins of NCI-H295R. EN = Nuclear Extract, EC = Cytosolic Extract, Figure S6: Trabectedin exposure affects the subcellular localization of β-catenin in NCIH295R cells.
