# Supplementary material for: Cytotoxic Effect of Trabectedin In Human Adrenocortical Carcinoma Cell Lines and Primary Cells
Source: Cancers (Basel). 2020 Apr 9;12(4):928. doi: 10.3390/cancers12040928 (PMC7226156; doi:10.3390/cancers12040928)
Supplement: Supplementary file 1 [file cancers-12-00928-s001.pdf]

Supplemental Materials

Cytotoxic Effect of Trabectedin In Human Adrenocortical Carcinoma Cell Lines and Primary Cells

Andrea Abate, Elisa Rossini, Sara A. Bonini, Martina Fragni, Deborah Cosentini, Guido A.M. Tiberio, Diego Benetti; Constanze Hantel, Marta Laganà, Salvatore Grisanti, Massimo Terzolo, Maurizio Memo, Alfredo Berruti and Sandra Sigala

1. Evaluation of DNA Fragmentation

NCI-H295R cells were treated with the IC_{50} value of trabectedin for 4 days. DNA was extracted from cells using Apoptotic DNA Ladder Kit (ThermoFisher, Waltham, USA) and processed according to the manufactured. The DNA was loaded on 1.2% agarose-gel, containing 0.5 µg/mL ethidium bromide. Ethidium bromide-stained DNA was visualized by transillumination with UV light and was photographed.

2. Double staining AO/EtBr

NCI–H295R cells were treated with the IC_{50} value of trabectedin for 4 days. A double staining with acridine orange (AO) and ethidium bromide (EtBr) was performed to visualize and quantify the number of viable, apoptotic and necrotic cells, as described in [49].

Cells were examined by a Zeiss LSM 510 META confocal laser-scanning microscope (Carl Zeiss AG, Germany). Several fields, randomly chosen, were digitalized and scored by using the NIH Image J software.

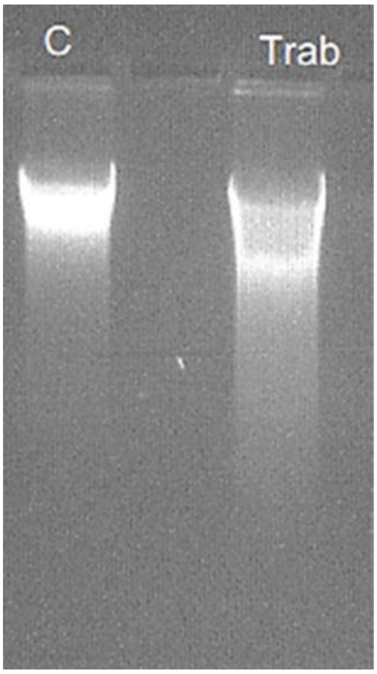

Figure S1. Effect of trabectedin on DNA integrity. NCI-H295R cells were treated with trabectedin at its IC_{50} for 4 days. DNA from treated and untreated cells was extracted and loaded on agarose-gel, containing ethidium bromide.

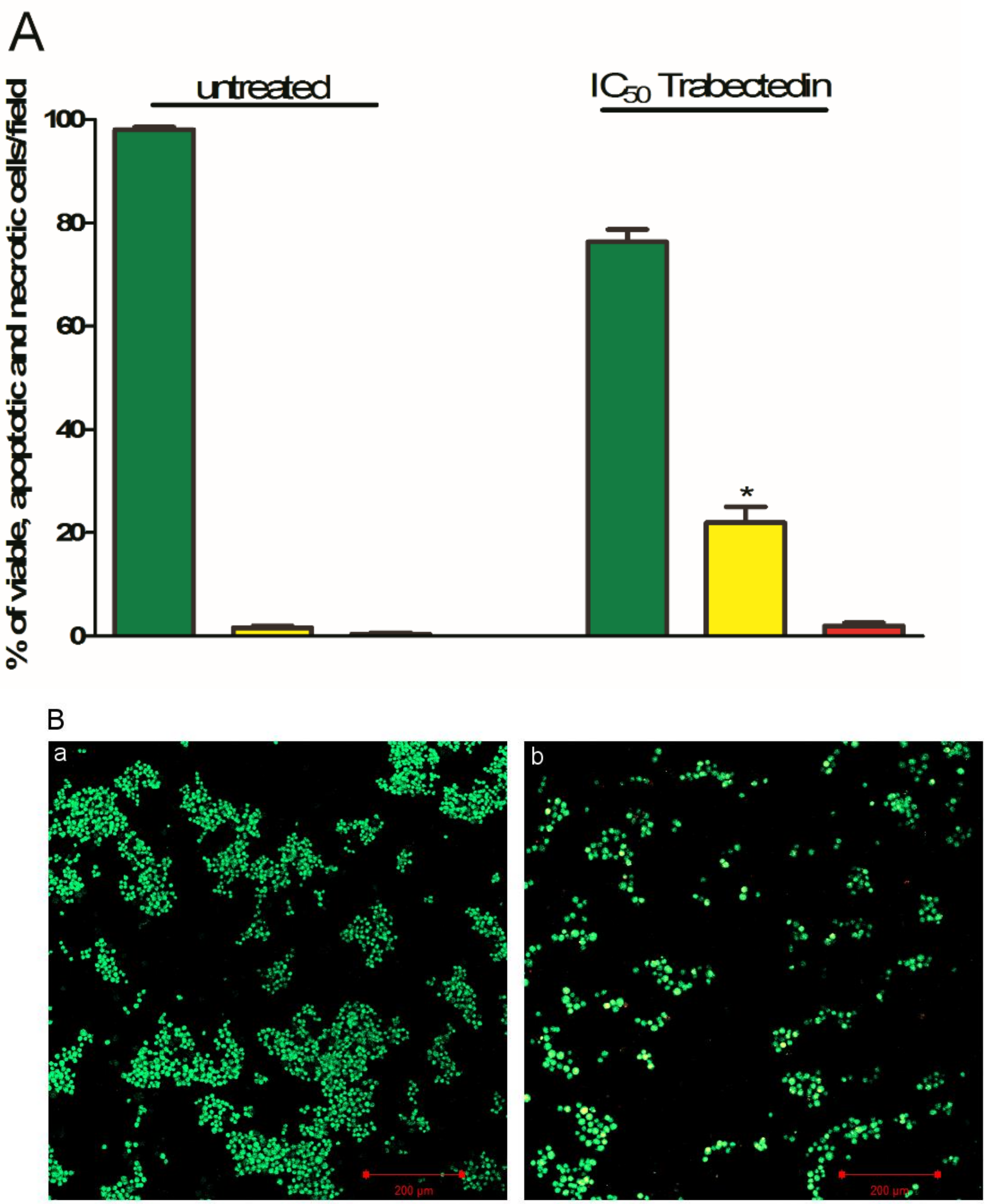

Figure S2. Trabectedin promoted apoptotic cell death in NCI-H295R cells.NCI-H295R cells were treated for 4 days with trabectedin at its IC_{50} and then stained with AO/EtBr. (**A**) Viable (green), apoptotic (yellow) and necrotic (red) cells were scored under a confocal laser-scanning microscope. Bars represent the percentage of each cell colour vs the total number of cell/field. * $p < 0.01$ vs. untreated apoptotic (yellow) cells. (**B**) The images were representative of several acquired fields (**a**) untreated and (**b**) trabectedin-treated cells. Magnification, 10×.

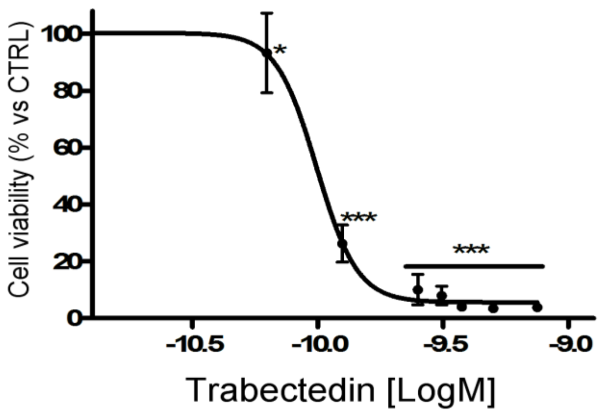

A

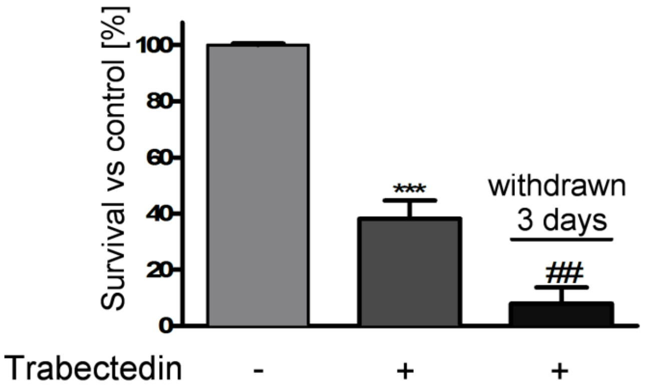

B

Figure S3. Cytotoxic effect of trabectedin on SW13 cell line. (**A**) SW13 cells were treated for 3 days with increasing concentrations (0.0625–0.75 nM) of trabectedin. (**B**) SW13 cells were treated with 0.09 nM trabectedin for 3 days, then trabectedin was withdrawn from medium and cells were kept in culture for further 3 days. Cell viability was analyzed by MTT assay. Results are expressed as percent of viable cells vs untreated cells ± SD; * $p < 0.01$, *** $p < 0.0001$, $^{\#\#\#}$ $p < 0.0001$ vs trabectedin-treated cells; $^{\#\#}$ $p < 0.01$ vs trabectedin-treated cells.

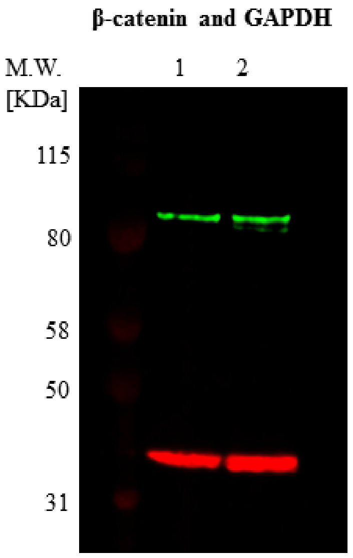

1: Ctrl
2: IC_{50}

Lane n°	β-catenin intenity	GAPDH intensity	β-catenin/ GAPDH	Ratio
1	0.1925	0.2640	0.7292	100
2	0.2570	0.3530	0.7280	99.83

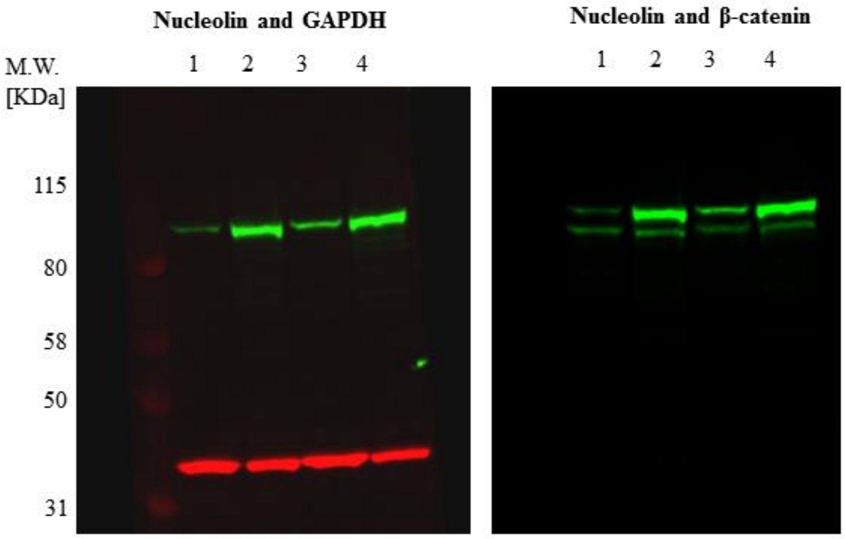

1: Ctrl EC (Cytosolic Extract)
2: Ctrl EN (Nuclear Extract)
3: IC_{50} EC
4: IC_{50} EN

Lane n°	β-catenin intensity	Nucleolin intensity	β-catenin/ nucleolin	Ratio
2	0.0770	0.1350	0.5704	100
4	0.0560	0.1925	0.2909	51

Figure S4. Whole western blots of Figure 4.

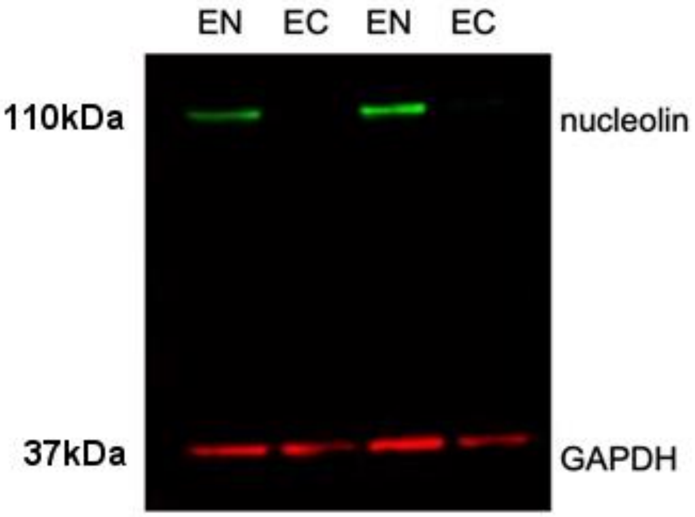

Figure S5. Representative figure of subfractions proteins of NCI-H295R. EN = Nuclear Extract, EC = Cytosolic Extract.

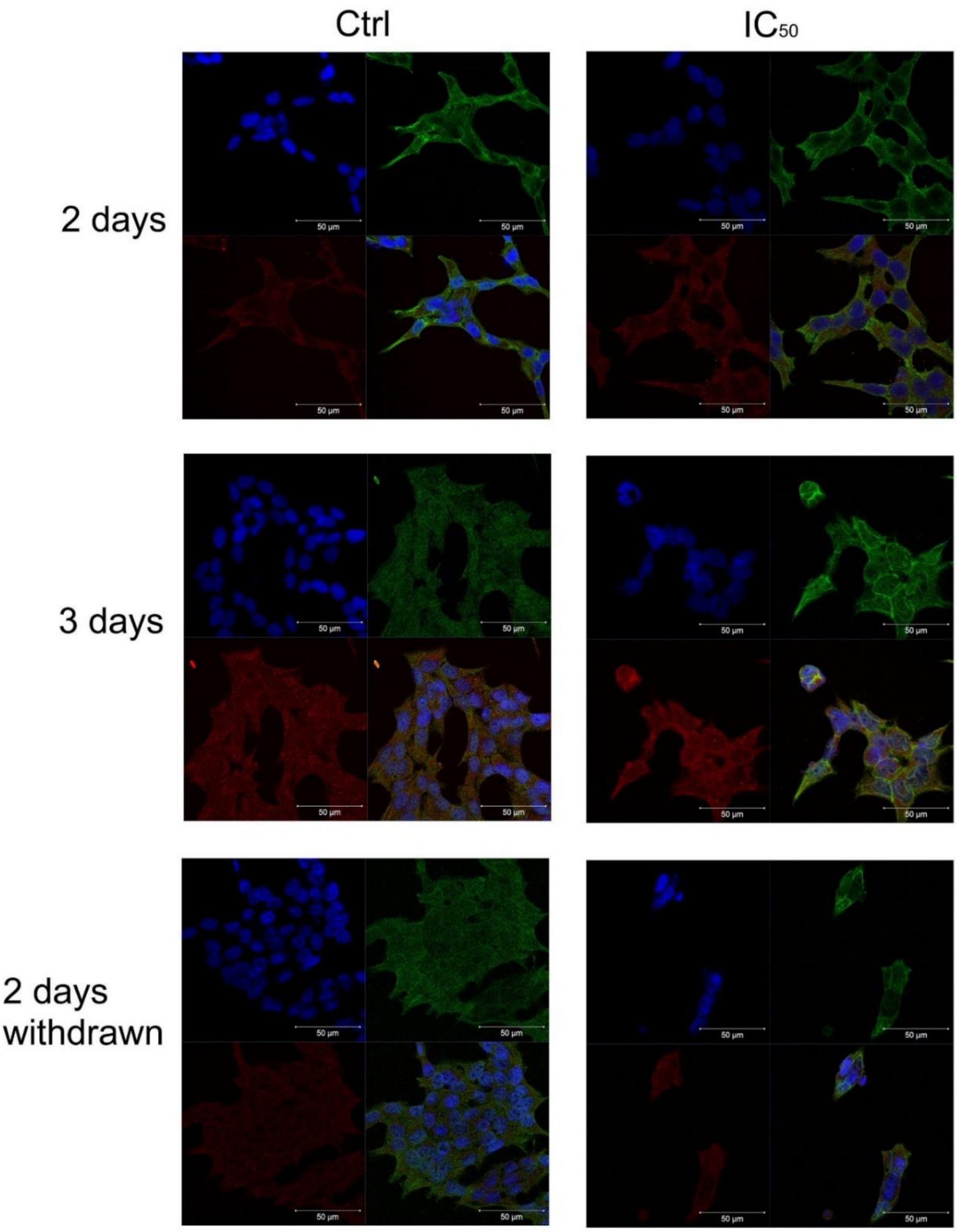

Figure S6. Trabectedin exposure affects the subcellular localization of β-catenin in NCIH295R cells. Cells were treated with 0.15 nM trabectedin for 2, 3 and 4 days. After 4 days of treatment, trabectedin was withdrawn from medium and cells were kept in culture for further 2 days. Untreated and trabectedin treated cells were analyzed for β-catenin localization following by incubation with Hoechst for nuclear staining. For each panel, the top-left insert: Hoechst; top-right insert; β-catenin; bottom-left insert: constitutive proteasome subunit PSMB5; bottom-right insert: merge. The scale bar of 50 μm is automatically inserted by the software ZEN Black.

© 2020 by the authors. Licensee MDPI, Basel, Switzerland. This article is an open access article distributed under the terms and conditions of the Creative Commons Attribution (CC BY) license (http://creativecommons.org/licenses/by/4.0/).
